# Enhancing Tumor Targeted Therapy: The Role of iRGD Peptide in Advanced Drug Delivery Systems

**DOI:** 10.3390/cancers16223768

**Published:** 2024-11-08

**Authors:** Dragana Nikitovic, Ekaterina Kukovyakina, Aikaterini Berdiaki, Alexandros Tzanakakis, Anna Luss, Elizaveta Vlaskina, Anne Yagolovich, Aristides Tsatsakis, Andrey Kuskov

**Affiliations:** 1 Department of Histology-Embryology, Medical School, University of Crete, 71003 Heraklion, Greece; berdiaki@uoc.gr; 2 Department of Technology of Chemical Pharmaceutical and Cosmetic Products, D. Mendeleev University of Chemical Technology of Russia, 125047 Moscow, Russia; kukovyakina.e.v@muctr.ru (E.K.); luss.a.l@muctr.ru (A.L.); vlaskina.e.r@muctr.ru (E.V.); kuskov.a.n@muctr.ru (A.K.); 3 School of Electrical and Computer Engineering, National Technical University of Athens, 15780 Athens, Greece; el18431@mail.ntua.gr; 4 Faculty of Biology, Lomonosov Moscow State University, 119234 Moscow, Russia; yagolovichav@my.msu.ru; 5 Forensic Medicine Department, Medical School, University of Crete, 71003 Heraklion, Greece; tsatsaka@uoc.gr

**Keywords:** iRGD, nanocarriers, cancer therapy, cell-penetrating peptides (CPPs), tumor microenvironment

## Abstract

Chemotherapy remains the primary therapeutic approach in treating cancer. The tumor microenvironment (TME) is the complex network surrounding tumor cells, comprising various cell types, such as immune cells, fibroblasts, and endothelial cells, as well as ECM components, blood vessels, and signaling molecules. The often stiff and dense network of the TME interacts dynamically with tumor cells, influencing cancer growth, immune response, metastasis, and resistance to therapy. The effectiveness of the treatment of solid tumors is frequently reduced due to the poor penetration of the drug, which leads to attaining concentrations below the therapeutic levels at the site. Cell-penetrating peptides (CPPs) present a promising approach that improves the internalization of therapeutic agents. CPPs, which are short amino acid sequences, exhibit a high ability to pass cell membranes, enabling them to deliver drugs efficiently with minimal toxicity. Specifically, the iRGD peptide, a member of CPPs, is notable for its capacity to deeply penetrate tumor tissues by binding simultaneously integrins ανβ3/ανβ5 and neuropilin receptors. Indeed, ανβ3/ανβ5 integrins are characteristically expressed by tumor cells, which allows the iRGD peptide to home onto tumor cells. Notably, the respective dual-receptor targeting mechanism considerably increases the permeability of blood vessels in tumors, enabling an efficient delivery of co-administered drugs or nanoparticles into the tumor mass. Therefore, the iRGD peptide facilitates deeper drug penetration and improves the efficacy of co-administered therapies. Distinctively, we will focus on the iRGD mechanism of action, drug delivery systems and their application, and deliberate future perspectives in developing iRGD-conjugated therapeutics. In summary, this review discusses the potential of iRGD in overcoming barriers to drug delivery in cancer to maximize treatment efficiency while minimizing side effects.

## Simple Summary

The effectiveness of chemotherapy, the primary cancer treatment, is often limited in solid tumors due to poor drug penetration, resulting in subtherapeutic concentrations. Cell-penetrating peptides (CPPs), short amino acid sequences that have the ability to enhance drug delivery with minimal toxicity, are emerging as a promising solution. The iRGD peptide, belonging to the CPP class, plays a crucial role in penetrating tumor tissues as it targets explicitly ανβ3/ανβ5 integrins and neuropilin receptors, established to be commonly expressed in tumor cells. This dual-receptor mechanism improves tumor blood vessel permeability, allowing deeper and more efficient drug or nanoparticle delivery. This review highlights the iRGD mechanism and drug delivery systems and emphasizes future developments in iRGD-conjugated therapeutics to enhance cancer treatment efficacy while reducing side effects.

## 1. Introduction

Chemotherapy, the most common cancer treatment, needs to address various challenges. Thus, its utilization is correlated to severe side effects, including toxicity in healthy cells, drug resistance, long-term health consequences, and high costs. Moreover, chemotherapeutics often exhibit limited penetration into tumor tissue, which is directly correlated to reduced efficacy. The tumor microenvironment (TME) is the complex ecosystem surrounding tumor cells, comprising various cell types, such as immune cells, fibroblasts, and endothelial cells, as well as ECM components, blood vessels, and signaling molecules. The TME interacts dynamically with tumor cells, influencing cancer growth, immune response, metastasis, and resistance to therapy The subtherapeutic drug concentrations at the tumor site allow further tumor growth and dissemination. Therefore, improving drug penetration and distribution within solid tumors is crucial to maximizing anticancer treatment effectiveness [1].

Cell-penetrating peptides (CPPs) have been developed for drug delivery applications. CPPs are short sequences of amino acids with an inherent ability to traverse cellular membranes and facilitate the internalization of various molecules, from small fluorophores to large proteins or DNA [2,3]. Several factors, including amino acid composition, structure, concentration, membrane lipids’ composition, and the cargo’s physicochemical properties, influence the translocation of CPPs. Research indicates that CPPs use both endocytic and non-endocytic pathways to enter cells, mostly without damaging membrane integrity [2,3]. The first discovered CPP was the TAT peptide in 1988, derived from a transcription activator protein encoded by the human immunodeficiency virus, capable of penetrating the cell membrane [4]. Further studies showed that modification of liposomes with a TAT peptide facilitated their delivery to various tumor cell lines [5]. A wide variety of CPPs have been discovered, which are classified by origin, physicochemical properties, structural characteristics, and application (extensively reviewed in [6]). For example, CPPs can be classified by origin as derived from natural (TAT, penetration, pVEG, etc.), synthetic (R8, MAP, etc.), and chimeric (MPG, CADY, etc.) proteins [7].

The main obstacles preventing the introduction of CPPs into clinical practice are their low selectivity for target versus non-target cells, rapid blood clearance, and enzymatic degradation under physiological conditions [6]. Various approaches are used to overcome these hindrances. For example, to enhance the recognition of tumor cells, CPPs based on the integrin αvβ3/5-specific RGD (arginine–glycine–aspartic acid) and cell-adhesion motif NGR (asparagine–glycine–arginine) sequences are being developed [8]. Other modifications include peptide cyclization, using L- instead of D-amino acids, and conjugation with nanocarriers [6,9].

The iRGD peptide is a novel tumor-penetrating peptide belonging to the CPP class The iRGD peptide, which stands for “internalizing RGD”, is derived from a sequence of amino acids that includes the RGD (arginine–glycine–aspartic acid) motif, known for its affinity to integrins expressed on the surface of tumor endothelial cells [10,11]. What sets iRGD apart from other RGD peptides is its additional C-end Rule (CendR) motif, which activates after proteolytic cleavage within the tumor microenvironment [10]. The CendR motif binds to neuropilin-1 and -2 (NRP-1/2) receptors upon activation, enhancing tissue penetration. The nanodelivery systems functionalized with the iRGD peptide have unique properties that provide additional benefits. Compared with other RGD-based CPPs, iRGD peptide can penetrate cells better, accumulate inside and around tumor vessels and in the tumor parenchyma, and spread faster and more efficiently in tumor tissues [11,12]. Next is the cyclic form of iRGD, which protects it from enzymatic degradation in vivo. Finally, functionalizing nanocarriers with iRGD, which endows tumor-targeting properties, provides the latter with increased plasma stability and, consequently, lower therapeutic doses required. Together with high tumor selectivity, these impacts reduced off-target effects and lowered immunogenicity risks.

CPPs are emerging as versatile tools in drug development and nanomedicine, offering a range of benefits. Their biocompatibility, membrane permeability, specificity, ease of synthesis, and stability make them highly promising for selective drug delivery. Their vulnerability to enzymatic degradation is a limitation that can be overcome through chemical modifications such as cyclization, conjugation with other molecules, or incorporating D-amino acids to enhance proteolytic stability. The aim is to utilize CPPs to deliver therapeutic or diagnostic molecules specifically to pathological cells, like cancer cells, while sparing normal cells, thereby boosting drug efficiency and reducing side effects [3].

**Figure 1:**
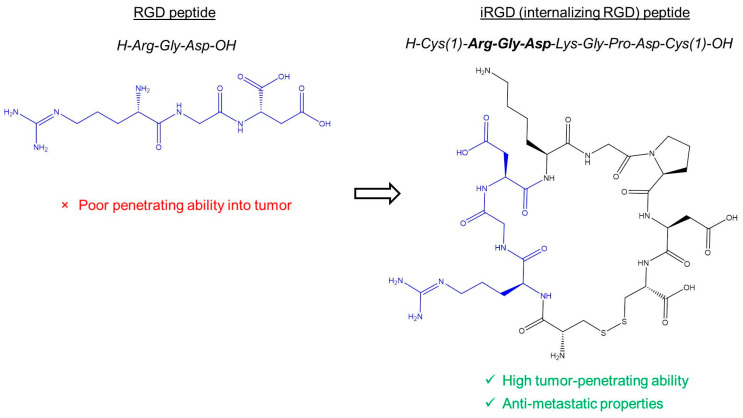
Chemical structure of RGD and iRGD peptides.

One of the primary challenges in solid tumors is their scant and insufficient vasculature, which results in poor tumor blood perfusion and restricts the delivery of chemotherapeutics to tumor cells [13,14]. The αvβ3 integrin-specific Arg-Gly-Asp (RGD) (cilengitide) can increase tumor blood diffusion. Thus, recently, doxorubicin (DOX) loaded smart liposome, MC-T-DOX, was synthesized to improve pancreatic cancer therapy by enhancing tumor blood flow and drug delivery. Loaded with DOX and a low-dose cilengitide—a peptide released in tumor vessels by MT1-MMP—MC-T-DOX increases blood perfusion, promoting its distribution and accumulation within the tumor. Heat-triggered DOX release then enhances tissue penetration, significantly boosting therapeutic effectiveness. This combined approach of vascular modulation and targeted delivery shows promise for treating hypoperfused tumors [15]. Another feature of cRGD peptides is that by explicitly targeting αvβ3 integrin, they stimulate angiogenesis at nanomolar concentrations by modulating the trafficking of αvβ3 integrin and vascular endothelial growth factor receptor-2, thereby promoting endothelial cell migration [16,17]. Therefore, the iRGD peptide can modify the blood perfusion within the tumor, thereby increasing the diffusion rate of small-molecule drugs. Its binding to NRP-1 facilitates extravasation, and it explicitly targets tumor cells overexpressing ανβ3/ανβ5 integrins while exhibiting low toxicity to normal cells [11]. The structure of the iRGD is depicted in Figure 1.

## 2. Tumor Tissue Structure, Remodeling, and Angiogenesis

### 2.1. Tumor Tissue Structure and Remodeling

In order to develop effective therapeutic strategies, the differences between healthy and tumor tissues need to be identified. These differences encompass a wide range of cellular and molecular characteristics, including changes in cellular morphology, behavior, genetic makeup, and interactions with the surrounding microenvironment.

Tissues consist of various cell types supported by the complex and intricate network of extracellular matrix (ECM) components. The ECM can be classified into the interstitial matrix and the basement membrane. The three-dimensional network of the interstitial matrix in tumors supports malignant cells, linking them with stromal cells and modulating their abilities to proliferate, migrate, and invade. It comprises, among others, collagens, fibronectin, proteoglycans, glycosaminoglycans, and elastin, which vary by tissue. The tumor matrix exhibits significant remodeling during carcinogenesis, affecting cell signaling, functions, and tumor progression. The basement membrane is a highly organized component of the ECM. It is a dense sheet, mainly composed of highly organized collagens and laminins, interconnected by proteins like heparan sulfate proteoglycans (HSPGs) and nidogen. The basement membrane significantly prevents in situ carcinoma from invading adjacent tissues [18,19].

Healthy tissues consist of cells with uniform morphology, organized structure, and specific functions. Conversely, tumor tissues are marked by cellular heterogeneity, disorganization, and abnormal morphology. Cancer cells often exhibit pleomorphism, which involves variations in size and shape and an increased nuclear-to-cytoplasmic ratio. These morphological changes reflect the underlying genetic and epigenetic alterations driving the malignant phenotype. Moreover, the TME exhibits a strongly remodeled ECM [19,20]. Importantly, tumor tissues are commonly denser and exhibit discrete mechanical properties, among other stiffer ECMs correlated to disease progression [21]. Therefore, chemotherapeutic drugs encounter denser ECMs, attenuating diffusion and further limiting their efficiency.

The interactions among these microenvironment components are vital in determining cancer progression [22,23]. The expression of ECM constituents is modulated in cancer and varies between different cancer types, affecting cell function and cancer development. Indeed, changes in specific ECM components [24] have been proposed as markers for disease progression and therapeutic targets [25,26]. Indeed, ECM molecules, such as fibronectin and vitronectin, which bear the tripeptide sequence RGD, exhibit varying expression in cancer cells [27]. Notably, fibronectin expression has been correlated to cancer cell chemoresistance [27], suggesting that it can be utilized as a valid therapeutic target [28].

The TME or tumor stroma poses a significant challenge to the efficient distribution of chemotherapeutic agents within tumor tissues, making it one of the primary obstacles in antitumor therapy. Thus, dense ECM, high interstitial pressure, and abnormal vasculature create a rigid, compact structure that restricts drug penetration and nutrient delivery to the tumor core [29]. Indeed, the modulation of the tumor ECM stiffness can affect chemotherapeutic agent delivery [21]. The ECM influences drug response primarily because drug distribution in tumor tissues occurs via diffusion. Solid tumors with a dense ECM exhibit hindered drug transport [30,31]. Thus, lysyl oxidase isoenzymes (LOX and LOXL1-4) stabilize collagen networks and significantly contribute to cancer progression. It was demonstrated that LOX and LOXL2 overexpression hampers doxorubicin diffusion in tumor spheroids, but this effect was reversed by inhibiting lysyl oxidase with 2-aminopropionitrile [32]. Notably, lysyl oxidases can regulate VEGF-A expression by oxidizing the PDGFR extracellular domain and downstream signaling [33]. Indeed, increased LOXL2 in tumors promotes endothelial invasion, a critical step in neo-angiogenesis, by enhancing endothelial cell motility [34].

Hypoxia and acidic conditions, also designated as chemical barriers, in the TME impair immune cell function, enhance tumor evasion, and reduce the efficacy of therapies that rely on oxygen, like radiation therapy [35].

Furthermore, immune-suppressive cells (e.g., regulatory T cells, myeloid-derived suppressor cells, and tumor-associated macrophages) create an immunosuppressive environment that limits immune cell activity and allows tumors to evade immune detection. This feature of the TME has been characterized as a biological barrier [36]. Moreover, altered signaling pathways and overexpression of efflux pumps, denominated as molecular barriers, can reduce drug efficacy, enhance resistance, and promote cancer cell survival and proliferation [37]. Due to challenges presented by the intrinsic properties of TME, it is of utmost importance to develop targeted and efficient therapeutic approaches that are the least toxic to normal tissue.

### 2.2. Tumor Angiogenesis

One of the hallmarks of cancer is its ability to form new blood vessels supporting the viability and growth of tumor cells. Thus, when a tumor reaches a volume of approximately 2 mm^3^, increased interstitial pressure is evident, attenuating the diffusion of essential metabolites and nutrients. Therefore, tumor tissues exhibit cellular hypoxia at an early stage of development [38]. The deficiency of oxygen, under hypoxia, interferes with the normal physiological metabolism of cells, triggering a series of specific response mechanisms in the organism to adapt to the low oxygen condition. Indeed, hypoxia enhances the levels of hypoxia-inducible factor (HIF), consisting of HIF-1α and HIF-1β, which together activate hypoxic response elements (HREs), facilitating the production of proangiogenic proteins like vascular endothelial growth factor (VEGF), platelet-derived growth factor (PDGF), and tumor necrosis factor-α (TNF-α) [39]. The hypoxic environment induces the sprouting of new blood vessels from existing ones, supplying the tumor cells with the necessary oxygen and nutrients for survival and proliferation [39]. Initially, pericytes are removed from existing vessels, followed by the endothelial cell basement membrane and ECM degradation, performed mainly by matrix metalloproteinases (MMPs). In continuation, endothelial cells are induced to proliferate and migrate to form unstable microvessels. Under the influence of the cytokines released due to hypoxia, the adjacent mesenchymal cells differentiate into pericytes and are integrated into the endothelial tubes. In this manner, new vessels are stabilized, and tumor blood flow is established [38].

Before the initiation of angiogenesis, during the “dormancy state”, the tumor grows slowly, remaining asymptomatic and nonmetastatic. The cancer grows rapidly after the initiation of the angiogenic switch and the development of the blood vessel network [40].

**Figure 2:**
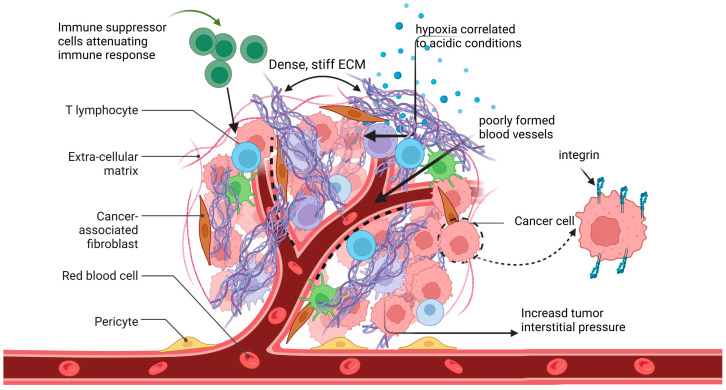
Tumor microenvironment. The TME consists of tumor and stromal cells, including lymphocytes, cancer-associated fibroblasts, and immune suppressor cells, surrounded by a dense, stiff, intensely remodeled ECM. It is characterized by hypoxia, acidic conditions, increased interstitial pressure, and intense angiogenesis, which results in poorly formed blood vessels. These physical, chemical, and biological features present considerable drug penetration barriers. Cancer cells exhibit discrete integrin expression compared to healthy cells. Created in BioRender. Nikitovic, D. (2024), BioRender.com/l29i302 (accessed on 30 October 2024).

The newly formed vessels are poorly constructed and exhibit higher permeability than normal vessels. The outcome is plasma leakage into the interstitial spaces, increasing interstitial fluid pressure. As a result, vessels may collapse, reducing the supply of oxygen and nutrients and attenuating the delivery of therapy agents [41]. Defective tumor angiogenesis also facilitates tumor cell transmigration across the endothelial barrier, aiding the spread of malignant cells throughout the body. Additionally, HIF-1α induces the expression of MMPs such as MMP-2 and MMP-9, which degrade the ECM and assist in tumor cell migration. Other targets of increased HIF-1α expression include membrane receptors like integrins [42]. The targeting of integrins expressed specifically by tumor endothelial cells enormously facilitates the delivery of chemotherapeutic agents. Consequently, aiming the vasculature is essential for effective tumor diagnosis and treatment [43]. A schematic depiction of the TME is presented in Figure 2.

### 2.3. Integrins

Integrins are heterodimeric transmembrane receptors that connect the extracellular matrix (ECM) to the cytoskeleton, enabling cell–cell and cell–ECM interactions. They consist of an α-subunit and a β-subunit, forming 24 unique αβ heterodimers in mammals. These receptors are activated by binding to ECM components like collagens and fibronectin, initiating intracellular signaling pathways involving kinases such as FAK, MAPK, and ERK. In endothelial cells, integrins also interact with the VEGF/VEGFR2 axis, linking them to the regulation of angiogenesis [44,45,46,47,48]. Integrins are a class of cell membrane receptors that can transmit mechanical forces across the cell membrane, influence intracellular signaling pathways, and facilitate cell adhesion to the ECM [44,45,46]. The resulting triggering of intracellular signaling enables integrins to regulate vital cell function [47]. For example, they are essential for cell motility, allowing tumor cells to detach, invade the ECM, transmigrate through the endothelium, and enter the blood or lymphatic vessels, disseminating to distant metastasis sites [48].

**Figure 3:**
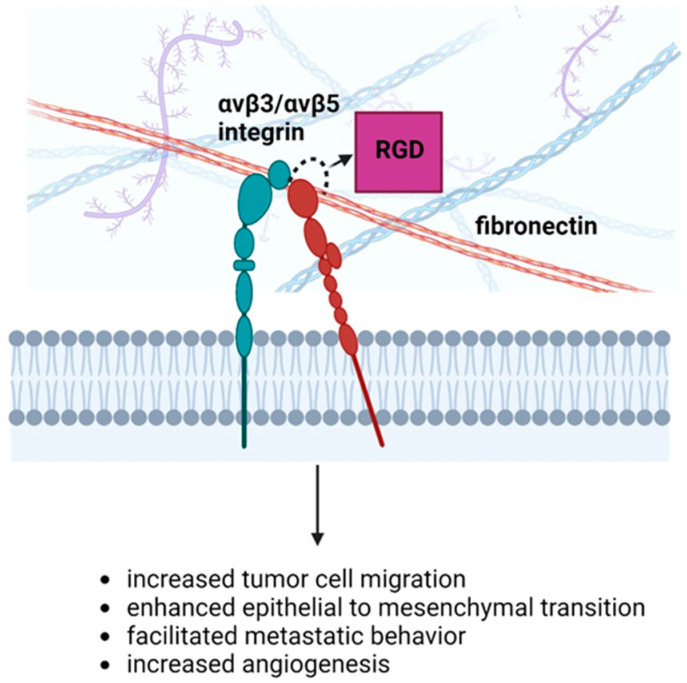
The roles of ανβ3/ανβ5 in the facilitation of carcinogenesis. Created in BioRender. Nikitovic, D. (2024), BioRender.com/c83e444 (accessed on 2 October 2024).

Integrins members α5β1, α2β1, α6β1, αvβ5, α5β3, and especially αvβ3 play key roles in tumorigenesis and angiogenesis. The αvβ3 integrin binds to RGD motifs in ECM proteins like fibronectin and vitronectin, enhancing cell motility and metastasis. Recently, it was shown in breast cancer that hypoxia activates αvβ3 integrin translationally, which in turn triggers the epithelial–mesenchymal transition program, cell migration, and increased metastatic behavior [49]. It was shown in glioblastoma that angiogenesis can also be driven by the direct interaction between brain tumor cells, including those with cancer stem-like properties (CSCs), and endothelial cells. In vitro studies have shown that this interaction is mediated by the binding of integrin αvβ3 on ECs to the RGD-peptide in L1CAM on CSCs [50]. The roles of ανβ3/ανβ5 integrins in cancer are depicted in Figure 3.

Notably, αvβ3 is highly expressed on angiogenic endothelial cells and several types of tumor cells but exhibits low expression on resting endothelial cells in normal tissues [51]. Likewise, a high expression of ανβ5 integrin has been shown in various cancers, including glioblastomas and breast and lung cancer cells. A high specific affinity between the RGD motif and αvβ3/avβ5 has been established. Elevated expression of ανβ3/ανβ5 by tumor cells and cancer endothelial cells correlated to disease progression in combination with these integrins’ low expression by normal cells characterize them as a highly suitable therapy target [52,53].

Tumor angiogenesis, the process of forming new blood vessels within tumors, is tightly regulated by integrins, facilitating endothelial cell adhesion and migration within the tumor microenvironment. Integrins interact with the ECM, promoting structural remodeling of tumor tissue to support vessel growth and invasion. iRGD specifically targets integrins in the tumor vasculature, enhancing drug delivery into tumor tissue by increasing vascular permeability and improving access to the remodeled tumor structure. This makes iRGD a powerful tool for directing therapies to highly vascularized, integrin-expressing tumors. Indeed, the iRGD peptide has been described as a groundbreaking agent in the fight against cancer, particularly for its ability to reduce cancer angiogenesis and regulate cell migration across the endothelium. Below, facets of iRGD’s characteristics and therapeutic applications will be discussed.

## 3. iRGD Cell Permeabilization Mechanism

The internalized RGD peptide, a cell-penetrating peptide, has a unique C-end Rule (CendR) motif, which activates after proteolytic cleavage within the tumor microenvironment, activating endocytosis [4].

iRGD was identified by Ruoslahti et al. as a nanopeptide with remarkable tumor-homing properties [54], increasing tumor penetration and effectiveness of chemotherapeutics [55,56,57,58,59]. Internalized arginyl–glycyl–aspartic acid cyclic peptide, or iRGD, is a nine-amino-acid cyclic peptide (CRGDKGPDC) containing two disulfide bonds that target tumors with high specificity and penetrative ability. The mechanism of cell permeabilization involves the iRGD (CRGD[K/R]GP[D/E]C) peptide binding to integrin isoforms. Then, iRGD is proteolytically cleaved to produce CRGD/K and exposes the cryptic CendR motif (R/KXXR/K) at the C-terminus arginine or lysine residues. Then, the CendR motif (also known as the C-terminal rule) binds to neuropilin-1 (NRP-1) and subsequently triggers active endocytosis to internalize iRGD and the associated cargo [60,61].

### 3.1. Integrin Binding

It is widely established that integrins are highly expressed by tumor cells and the endothelial cells of feeding vessels, with a lower expression level on the blood vessel cells of normal tissues [62,63]. Integrin expression can be modulated by the cell type and tissue microenvironment [64,65]. Eight integrin isoforms interact with the RGD motif of the ECM proteins (v1,v3,v5,v6,v8,5v1, 8v1, and IIb3) [66]. The importance of integrin v3 in cancer was the first to be identified [67]. It was presented to be highly expressed in different cancers, like gastric cancer, glioma, non-small cell lung cancer, pancreatic cancer, and prostate cancer [60]. The role of the v5 isoform, which was found to be crucial in regulating the growth of several cancer types, was also investigated [60].

iRGD was initially presented to interact with αvβ3 and αvβ5 integrin isoforms. Fibroblasts surrounding cancer cells produce several ECM components, including collagen, cytokines, and growth factors, like transforming growth factor-β (TGF-β) [68]. TGF-β induces expression of the β5 integrin subunit that dimerizes with the αv subunit, forming the αvβ5 integrin [44,69,70]. Avβ5 integrin, in turn, interacts directly with the RGD motif, regulating cell functions like adhesion, migration, and survival [71,72]. Research in pancreatic cancer presented that β5 integrin expression is essential for the tumor-penetrating actions of iRGD in mice [58]. More specifically, they described the importance of TGF-β, produced by cancer-associated fibroblasts and epithelial cancer cells, in the role of β5 integrin in tumor development—furthermore, the αvβ5 integrin changes angiogenesis through the FAK–steroid receptor coactivator pathway [73]. The latter was also mediated by αvβ3 integrin activity, modulating the p21-activated kinase signaling pathway.

A recent study by D’amore et al. (2023) [74] further investigated iRGD’s binding mechanism to integrins *α*v*β*3 and *α*v*β*5 and presented its interaction with an additional isoform, *α*v*β*6. *A*v*β*6 regulates cell growth, migration, and invasion in several cancers, including pancreatic ductal adenocarcinoma. iRGD was described as a valuable tool in pancreatic ductal adenocarcinoma for administering paclitaxel and gemcitabine [75,76]. Using advanced metadynamics simulations in an aqueous environment, iRGD formed a peculiar horseshoe-like conformation in order to perform interactions with integrin isoforms. The peptide’s activation mechanism relied on the proteolytic cleavage of its Lys5−Gly6 bond with integrin and the following release of the internalizing CendR motif. In addition, experiments presented that the potency and selectivity affinity of iRGD was as follows: αvβ3 ≥ αvβ5 > αvβ6. In particular, the horseshoe-like shape that formed assists the peptide in fitting the peculiar features of the integrins’ SDL cleft and seems essential to achieve nanomolar binding affinity [74]. Interestingly, they also described possible ways to fine-tune the affinity/selectivity of iRGD toward each integrin isoform, aiming to design new peptides potentially able to recognize the newly presented anticancer target, αvβ6.

### 3.2. Proteolytic Cleavage

Following the iRGD binding to the integrin receptors [12], a proteolytic cleavage occurs at the Lys5−Gly6 bond that permits the exposure and release of the cryptic C terminal CRGDK sequence (CendR motif) and a protease recognition site. The CendR motif is thereafter recognized by NRP-1, a tyrosine kinase co-receptor important in angiogenesis, cell migration, and invasion [77].

#### Neuropilin-1 (NRP-1) Binding and Enhanced Permeability

The binding of the CendR motif to NRP-1 initiates the internalization of the complex and mediates the resulting effects of iRGD [61]. The resulting CRGDK fragment from the proteolytic cleavage has a high binding affinity to NRP-1 compared to integrins [60]. This shift in the binding of CRGDK from integrins to NRP-1 activates endocytosis and enables permeation [78,79]. In the case of other RGD peptide types, they bind only to integrins and not to NRP-1 and are accumulated inside and/or around tumor vessels. Importantly, unlike integrins that are expressed in specific cancer types, NRP-1 expression is enhanced in the majority of tumor types [12,54,80,81]. NRP-1, a transmembrane glycoprotein containing a short transmembrane domain, was initially described as a neural adhesion molecule [82]. It was found essential for neural crest migration and axon guidance during neural development [83]. Also, it has been shown to modulate vessel formation, excess capillary formation, and hemorrhaging in mice [84]. NRP-1 also acts as a co-receptor, enhancing the attachment of the vascular endothelial growth factor A (VEGF-A) to the VEGF receptor [80], a signaling pathway important in tumor angiogenesis [85].

**Figure 4:**
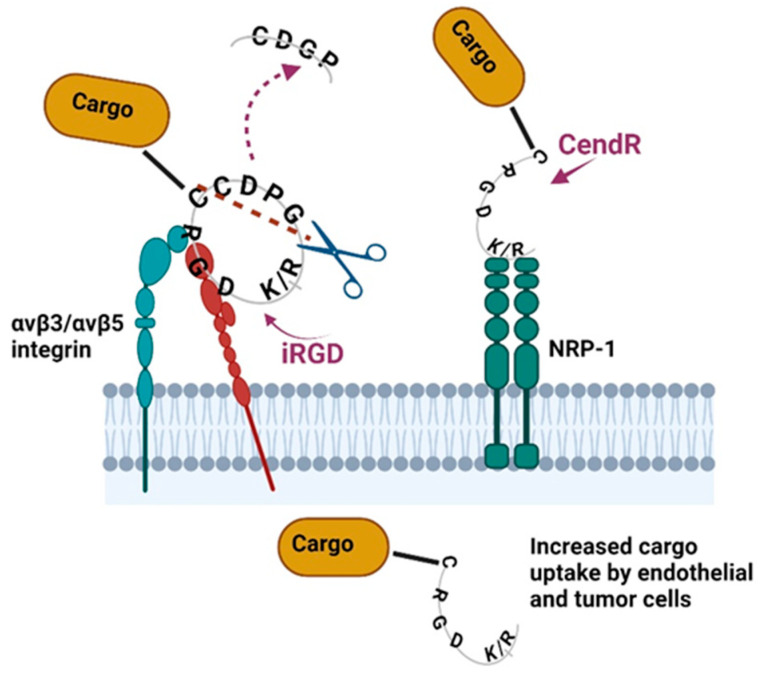
The iRGD mechanism of cargo delivery. The iRGD peptide carrying drug cargo binds to integrin β3/β5 through the RGD motif. This facilitates the cleavage of the CDGR sequence and enhances the binding of the remaining CendR motif carrying cargo to NRP1. CendR/NRP-1 binding improves cargo uptake by endothelial and tumor cells expressing β3/β5 integrin. Created in BioRender. Nikitovic, D. (2024), BioRender.com/y62t355 (accessed on 2 October 2024).

The CendR peptide primarily binds to NRP-1, but it can additionally bind to NRP-2, initiating endocytosis or exocytosis transport pathways. These CendR specific pathways, differ from the endocytosis mechanisms mediated by clathrin, caveolin, and dynamin [86,87,88]. The iRGD is taken up by cells through the NRP-1 receptor and actively transported across tissues, in contrast to the micropinocytosis mechanism, which is often receptor-independent and non-selective [86]. When iRGD is co-administered with substances/therapeutic components/cargo, it forms vehicles (grape-like clusters), allowing passage across the continuous vascular endothelium [89]. The diameter of these endocytic vesicles is approximately 200 nm and can contain substantial ECM fluid. Furthermore, CendR’s endocytosis is suggested to be modulated by nutrients and mTOR signaling [88]. Under conditions of nutritional deprivation, like an amino acid or glucose insufficiency, the action of the CendR pathway can be enhanced [86]. The mechanism of iRGD-functionalized drug cargo is presented in Figure 4.

In vitro, experiments support the inhibiting role of iRGD in tumor migration and enhancement of chemorepulsion based on a CendR/NRP-1 dependent mechanism [90]. Sugahara et al. (2015) showed that iRGD inhibits cell migration by adding this peptide in a mouse model and presenting a decrease in prostate cancer cell migration [90]. Interestingly, when the iRGD peptide was injected intravenously at a 4 μmol/kg concentration, the reduction in the metastatic ability of the cells was enhanced. Such experiments verified the critical role of iRGD in the targeted drug delivery of various therapeutic or diagnostic molecules directly toward cancer cells. More importantly, the binding of iRGD with these agents is without a need for a linker or chemical spacer [90].

Indeed, tumor interstitial pressure (TIP) is often elevated in tumors due to factors like dense ECM, abnormal vasculature, and poor lymphatic drainage. High TIP creates a physical barrier that hinders the delivery and distribution of therapeutic agents within the tumor, making effective drug penetration difficult. By binding to NRP-1, iRGD induces transient permeability in tumor vessels and surrounding ECM, effectively lowering resistance caused by high TIP. This reduction in TIP allows therapeutic agents to penetrate deeper into the tumor, bypassing physical barriers created by high interstitial pressure [78,79].

Each step described for the iRGD’s specific mechanism of cell permeabilization increases the tumor specificity of iRGD [11]. The cell-penetrating ability of iRGD proves better compared to other RGD peptides [91], and most importantly, iRGD has no adverse effect on healthy cells [92], making it an excellent candidate for targeted treatment applications.

## 4. Nanosized Drug Delivery Systems with iRGD and Their Application

To date, various nanoparticles with the conjugated iRGD peptide for the treatment of oncological diseases have been developed (Table 1): polymeric nanoparticles based on biocompatible polymers, such as polylactide-co-glycolide [56,93], polylactide [94], poly-ζ-caprolactone [95,96], liposomes (discussed in Section 4.2 of this article), exosomes [97,98,99], dendrimers [100,101], nanocages [102,103], and different inorganic nanoparticles, including iron oxide [104], gold [105,106], among others. An overview of the types of nanoparticles decorated with iRGD and their applications are summarized in Table 1.

### 4.1. Methods for the Modifications of Nanoparticles

iRGD is a tumor-penetrating peptide that could be crucial in treating cancer. Its unique ability to penetrate tumor tissues deeply establishes it as an outstanding candidate for targeted drug delivery. Most studies describe methods for attaching iRGD to the surface of nanosized particles, enhancing their tumor-penetrating capabilities, and improving the efficacy of cancer treatments.

There are many methods for coupling a peptide to a polymer. One of the most common reactions for coupling carboxyl and amine groups is conjugation via the EDC/NHS. EDC activates the carboxyl groups, forming an active intermediate O-acylisourea. To increase the stability of this compound, NHS or its water-soluble analog sulfo-NHS is used, which converts the intermediate into a sulfo-NHS ester. The amine-reactive intermediate then reacts with the primary amine groups via nucleophilic attack, forming an amide bond. The carbodiimide coupling method has been widely used to couple the iRGD peptide containing an NH_2_ group to a polymer with a COOH group. In Huang [117] and Pan [118] works, the peptide attached to DSPE-PEG2000–NHS to form DSPE-PEG2000-iRGD for further modification of the erythrocyte membrane [117] and extracellular vesicles [118]. It is worth noting that despite the efficiency of the reaction, EDC/NHS coupling is not a selective reaction; the bonding with the carrier can be carried out by any free NH2 groups of the peptide, which can lead to peptide inactivation. This reaction can also lead to peptide oligomerization, which can also negatively affect the activity of the peptide.

**Figure 5:**
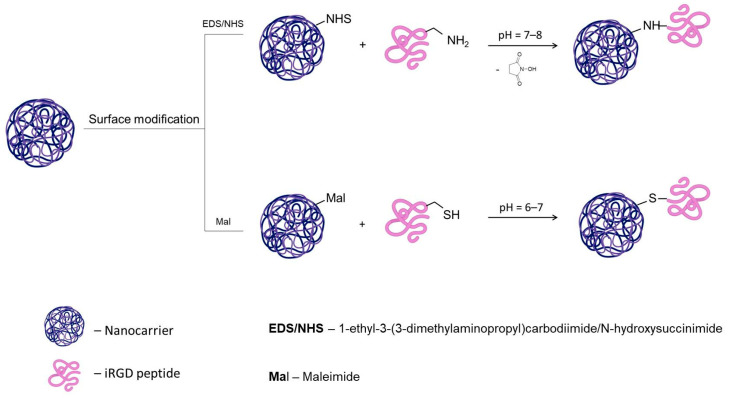
Mechanisms of iRGD conjugation.

Another standard method is the Michael reaction, which involves creating a bond between a nucleophile, a donor, and an α,β-unsaturated system. Of particular interest are maleimides due to the rapid reaction rate and their high reactivity of the C=C bond. This is due to the presence of two activating carbonyl groups in combination with ring-strain/bond angle distortion. Maleimides are also virtually invisible to functional groups other than thiols, allowing them to bind to cysteine selectively. This reaction also has disadvantages: with many cysteine residues, the reaction can occur at any of them, which can also affect the properties of the peptide. The second disadvantage is the absence of cysteine residues in the peptide, which must be added in the case of synthetic production of the peptide. The works [119,120,121,122] reflect the ability of the peptide to bind to the maleimide group and retain the capability to penetrate the tumor together with the loaded nanosized carrier. The essential characteristics of the EDC/NHS and maleamic conjugation are depicted in Figure 5.

**Figure 6:**
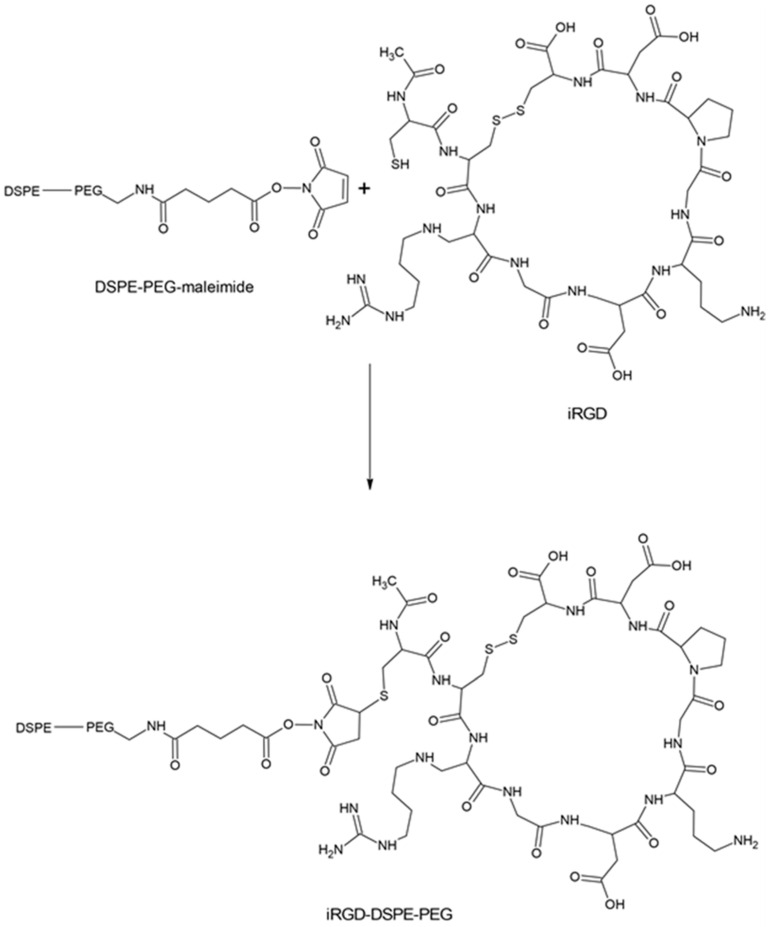
Attachment of the iRGD peptide to a pegylated lipid.

An established method to increase the nanoparticle delivery efficiency is the PEGylation of their surface. The specific coating protects against aggregation, opsonization, and phagocytosis, thereby prolonging the circulation time of nanoparticles in the bloodstream. PEG-modified with a maleimide group is often used for the rapid binding of particles and peptides. In works [94,112,113,123,124,125,126,127], a method for adding a peptide via a nucleophilic addition reaction of the cysteine sulfhydryl group of the peptide and the maleimide group of PEG is described (Figure 6).

It is also worth mentioning that in the articles [93,100], the peptide is attached to the maleimide group of PEG, and PEG itself is attached to the amino group of the polymer with the NHS ester group. Jin et al., in their work [128], describe the attachment of a peptide to nanoparticles using the MAL–PEG2000–NHS linker. The maleimide group reacted with the thiol group of iRGD at pH 7.1, and the hydroxysuccinimide group reacted with the amino group of β-Lactoglobulin.

In some cases, it is possible to attach a peptide using genetic engineering. Thus, Murata et al. [102] attached a peptide to the C-terminal region of the heat shock protein (HSP) cages using flexible linker fragments. Such nanocages with linkers of different lengths were prepared using the *Escherichia coli* protein expression system. The resulting composition showed the possibility of selective capture by cells with high expression of neuropilin-1 but not by cells with low expression of neuropilin-1.

Zhang et al. [129] created recombinant anti-EGFR-iRGD proteins in their work using the *Escherichia coli* protein expression system, which was attached to the surface of nanoparticles. Recombinant anti-EGFR-iRGD proteins were modified with N-succinimidyl palmitate, resulting in lipid-derivatized proteins. Then, using lipid bonds, the proteins were incorporated into RBCm vesicles modified with PLGA nanoparticles. Compared with nanoparticles without modification, the resulting composition showed better attachment and accumulation at the tumor site.

Another type of iRGD targeting differs from standard chemical coupling to the nanoparticle’s surface. In this case, exosomes, endogenous nanosized vesicles secreted by most cell types that can deliver chemotherapeutic drugs, are used. Immature dendritic cells (ImDCs) produce exosomes to reduce immunogenicity and toxicity. Tumor targeting is achieved by expressing a well-characterized exosomal membrane protein (Lamp2b) conjugated to the iRGD peptide. Immature DCs are transfected with a vector expressing iRGD–Lamp2b fusion proteins using the Lipofectamine transfection reagent. Exosomes are subsequently extracted from the cell culture medium, typically by centrifugation. In continuation, the incorporation of drugs or genetically engineered constructs occurs via electroporation, which creates pores in the lipid bilayer [99].

In [98], exosomes with the iRGD peptide on the surface were obtained, demonstrating highly efficient tumor cell targeting. siRNA encoding CPT1A (carnitine palmitoyltransferase 1A, a key enzyme in fatty acid oxidation) was loaded into iRGD exosomes for specific delivery of RNA to tumors to suppress fatty acid oxidation. Modified iRGD exosomes demonstrated significant inhibition of CPT1A in tumor tissues and showed the ability to reverse oxaliplatin resistance and inhibit tumor growth by inhibiting fatty acid oxidation in vivo.

In [97], iRGD exosomes showed highly efficient targeting and delivery of the anticancer drug doxorubicin to αv-integrin-positive breast cancer cells in vitro, as shown by flow cytometry and confocal imaging. In in vivo studies, the exosomes delivered doxorubicin specifically to tumor tissues after being administered intravenously, resulting in tumor growth inhibition without apparent toxicity.

### 4.2. iRGD Immobilized Liposomes

Liposomal nanocarriers are spherical vesicles with an aqueous core consisting of one or more lipid bilayers into which various therapeutic agents can be encapsulated, including peptides, small molecules, or nucleic acids. These characteristics of liposomal drugs endow them with significant advantages in cancer therapy; however, their clearance by the reticuloendothelial system and difficulty in targeting and penetrating tumors attenuate their efficacy. Liposomes can be modified with polyethylene glycol (PEG) to evade rapid clearance by the reticuloendothelial system to create a “stealth” effect, reducing macrophage recognition and prolonging circulation. Optimizing liposome size, charge, lipid composition, and incorporating targeting ligands or biomimetic coatings can also minimize RES uptake and improve bioavailability for drug delivery [130,131,132]. These challenges demonstrate the urgent need for further research and development in this field [133]. Active targeting enhances the specificity of drug-loaded nanoparticles (NPs) to tumors by modifying NPs with receptors or molecules highly expressed in tumor tissues, enabling receptor-mediated endocytosis [134].

One of the primary methods for conjugating liposomes with the iRGD peptide involves attaching iRGD to the DSPE-PEG2000 lipid incorporated in the liposome membrane. PEG lipid, widely used to form micelle-type nanoparticles for drug delivery, plays a vital role in this process. The attachment is achieved by inducing a reaction between the maleimide group of PEG lipid and cysteine-modified iRGD peptide, forming a male-mide–thiol covalent bond [135]. The decoration of liposomal nanocarriers with iRGD pep-tides significantly impacts their therapeutic efficacy. Indeed, this modification substantially enhances antitumor drug doxorubicin cytotoxicity on breast cancer cell lines and alters the pathway of nanosystem penetration into the cell. The identified shift from cavelin-dependent to clathrin-dependent endocytosis, followed by transport through early endosomes and lysosomes, allows more efficient delivery of doxorubicin encapsulated inside liposomes to the tumor in vivo. The modulations of the uptake mechanism significantly suppress tumor growth, underscoring the potential of iRGD-functionalization to enhance the therapeutic effectiveness of liposomal nanocarriers [120]. Another instance of improved doxorubicin efficacy is its delivery through iRGD peptide-modified sterically stable liposomes (iRGD-SSL-DOX) for melanoma treatment. iRGD-SSL-DOX liposomes can accumulate in the tumor tissue and exhibit increased antitumor activity in vivo compared to liposomes not modified with iRGD peptide. This study [136] demonstrated the potential of iRGD-modified liposomes for targeted drug delivery and imaging in cancer therapy. Interestingly, a separate study revealed that the combined administration of iRGD-SSL-DOX liposome and plain iRGD peptide did not enhance the antitumor effect of iRGD-SSL-DOX liposomes [136,137]. Moreover, loading various therapeutic agents into liposomes together with iRGD peptide conjugation on the liposome surface allows effective action on multiple targets in the organism. Thus, recombinant Newcastle disease virus expressing the dendritic cell chemokine MIP-3α (iNDV3α-LP) being immobilized on vessel-targeting, iRGD-functionalized liposome carriers induce tumor-specific cellular and humoral immunity, inhibits angiogenesis and suppresses the immunosuppressive tumor microenvironment [126]. Another example of iRGD peptide utilization is the respective functionalization of liposomes with encapsulated antisense oligonucleotides (ASOs) against the androgen receptor (AR) gene and its splice variants for the treatment of prostate cancer. Using iRGD-decorated liposomes is characterized by a significant knockdown efficiency of AR gene expression during such liposomal composition application in vitro. In prostate tumor models in vivo, iRGD liposomes with immobilized ASO, compared with free ASO, exhibit increased ASO accumulation in tumor tissue, correlated to a significant reduction in AR expression. This promising approach not only enhances ASO penetration but also suppresses the development of metastases in bone-metastatic prostate cancer models. The potential of iRGD-modified liposomes for treating various types of cancer is to be fully exploited in cancer therapy [125]. Pigment-epithelium-derived factor (PEDF) is a multifunctional protein with anti-angiogenic, antitumor, pro-apoptotic, and cardioprotective effects [138]. However, PEDF’s clinical use is limited by high costs, production challenges, and a short half-life [139]. An iRGD-functionalized long-circulating cationic liposome was developed for the targeted delivery of PEDF-DNA in the colorectal cancer (CRC) metastasis model. This application exhibited efficiency in attenuating invasion and migration and inducing pro-apoptotic effects of CRC cells in vitro. Furthermore, the utilization of the iRGD-decoration decreased the number of metastatic tumor nodules in the lung and increased the survival time in vivo [140].

In most cases, a single therapeutic molecule is loaded into liposomes. Still, recently, it was shown that loading several biologically active compounds can promote the manifestation of a synergistic antitumor effect. Thus, simultaneous encapsulation of curcumin and piperine in iRGD-modified liposomes showed potential synergistic cytotoxic effects dependent on exposure time and concentration. Furthermore, the proliferation and invasion of A549 lung tumor cells were suppressed. Notably, in vivo, such a nanosystem presented significant accumulation in lung tumor tissue and inhibited its growth [141]. A separate example of co-delivery is iRGD-peptide-modified liposomal nanoparticles load-ed with zinc phthalocyanine (ZnPc) as a hypoxia-enhancing photosensitizer and tirapa-zamine (TPZ) as a hypoxia-activating prodrug (iRGD@ZnPc + TPZ). Such a nanosystem promotes photodynamic therapy and chemotherapy under hypoxia, leading to enhanced glioblastoma tumor cell necrosis. Indeed, iRGD@ZnPc + TPZ liposomes can penetrate the blood-brain barrier (BBB) into the deep layers of gliomas and be retained in the tumor tissue. ZnPc, once internalized and radiated, depletes oxygen to produce reactive oxygen species, suppressing tumors and intensifying hypoxia to activate TPZ, thereby facilitating chemotherapy effects and inhibiting glioma growth. Utilizing these liposomes extended the survival time of mice with glioma exhibiting excellent biocompatibility with animal organisms in in vivo experiments [142].

Liposomal carriers of the iRGD peptide are not only a promising tool for the therapy of malignant neoplasms but also for their visualization. The potential of molecular imaging for early prediction of tumor progression by assessing biomarker levels is well-known. However, most techniques are limited in capturing complex biological processes. Ultrasound molecular imaging (UMI), using targeted microbubbles to bind specific markers, has been suggested for early disease diagnosis and monitoring [143]. Recently, the utilization of ανβ3-targeting liposomes increased the efficiency of visualization. A complex consisting of microbubbles conjugated with iRGD-peptide-modified liposomes and quantum dots encapsulated inside the liposomes (iRGD-QDLM) was obtained for dual-mode molecular visualization of tumor angiogenesis. With ultrasound treatment, iRGD-QDLM liposomes demonstrated the possibility of visualization of transvascular fluorescence by disrupting microbubbles with ultrasound and attaching to integrin αvβ3 on breast cancer cells. This novel approach creates new opportunities for tumor imaging. However, there are some limitations to this method. For example, non-specific interference signals from unmodified liposomes in the tumor affect the assessment of biomarker visualization on the surface of tumor cells due to the increased permeability and retention effect [144]. Another liposomal iRGD nanocarrier can perform dual-modality ultrasound/photoacoustic imaging in vitro and in vivo and selectively target liver cancer cells without affecting healthy hepatocytes. The specific nanocarrier penetrates tumor cells and releases the chemotherapeutic agent (10-hydroxy camptothecin) under the influence of low-intensity focused ultrasound (LIFU) irradiation. The combined action of iRGD-immobilized liposomes with LIFU irradiation exhibits a strong pro-apoptotic effect, promotes an increase in the anti-proliferation capacity of cells, and results in the death of most tumor cells in vitro. This nanosystem also demonstrated an increased therapeutic effect on tumors in vivo. Indeed, the decorated liposomes reached the tumor site faster than liposomes not modified with the iRGD peptide and did not accumulate in healthy internal organs [145].

However, despite the wide variety of liposomal carriers for iRGD peptides and their enhanced antitumor effect in vitro and in vivo studies, they have not yet reached the stage of clinical trials. This may be because modification of the liposome surface with iRGD peptide may have a negative impact on the stability, solubility, and pharmacokinetic properties of the final product, which may lead to undesirable side effects and a decrease in antitumor activity. In addition, it is challenging to select the optimal ligand density on the liposome surface since an increase in density above the optimal value may contribute to the aggregation of nanocarriers. In addition to the antitumor activity data, important information is the safety of iRGD liposomes for healthy organs and tissues, which is practically not available at the moment. Furthermore, amplifying laboratory methods for iRGD liposome development presents significant challenges for the pharmaceutical industry. Various factors must be considered, from the availability and cost of starting materials in large quantities to assuring drug loading efficiency, stability, sterilization, and consistency across batches.

### 4.3. iRGD Peptide Polymeric Nanocarriers

Polymeric nanocarriers with immobilized biologically active substances are a promising delivery strategy with the potential to facilitate drug delivery at both therapeutic and diagnostic levels. They offer many advantages over other types of carriers, such as improved pharmacokinetic parameters, increased biocompatibility and bioavailability, relatively simple production technology, and a wide range of drugs available for immobilization, including poorly soluble biologically active agents. The increased efficiency of such systems, especially the regulated and sustained release of the active compound from the polymeric system, coupled with the modification of the nanocarrier surface with a target ligand, opens up new possibilities for direct and efficient delivery of therapeutic agents to the tumor, thereby increasing their effectiveness [146].

For example, nanoparticles based on 6-maleimidocaproic acid loaded with paclitaxel and conjugated with thiolated iRGD peptide via Michael reaction were shown to be stable in phosphate-buffered saline (PBS) and fetal calf serum containing PBS. They were characterized by prolonged release of paclitaxel from the particle core. Also, they had increased cytotoxic activity compared to free paclitaxel in vitro and could accumulate in tumors in vivo [119]. Another example of polymeric nanocarriers with encapsulated paclitaxel is nanoparticles based on copolymers of poly(ε-caprolactone)-b-poly(N-vinylpyrrolidone) conjugated with iRGD peptide via a thiazolidine ring. They accumulated more effectively in the tumor and penetrated tumor tissues more efficiently compared to unmodified particles. Treatment with these nanocarriers significantly extended the life of mice with hepatocellular carcinoma [95].

Their surface is often coated with various agents to improve the pharmacological properties of nanoscaled carriers and impart them with specific characteristics. Chitosan is a natural cationic polysaccharide that is biocompatible, biodegradable, and low toxic, and it exhibits mucoadhesive properties [147]. Polymeric nanoparticles based on poly(lactide-co-glycolide) loaded with carmustine were coated with a chitosan shell modified with iRGD peptide by proton integration. The carmustine sensitizer (O^6^-benzyl guanine) was introduced into the chitosan layer. Being released from the shell first, O^6^-benzylguanine depletes O^6^-methylguanine-DNA-methyltransferase, increasing the sensitivity of tumor cells to carmustine, which is then released from the particle core. In addition, the chitosan coating changed the particle surface charge from negative to positive. As a result, positively charged iRGD-NPs can bind to anionic sites of brain capillaries, which facilitates the penetration of particles through the blood–brain barrier (BBB). iRGD-NPs exhibited significant cytotoxicity and cellular uptake in vitro and penetrated deeper tumor layers on 3D glioma spheroids compared to unmodified nanoparticles. In vivo experiments showed that iRGD-NPs penetrated the BBB and accumulated in brain tumors [107].

Coating nanoparticles with depolymerized chitosan has led to successful outcomes in in vivo experiments, providing reassurance about the potential of polymeric nanocarriers. Nanoparticles based on PLGA, loaded with camptothecin and modified with iRGD peptide, were prepared by forming a covalent bond between the sulfhydryl group of iRGD and the maleimide group of NHS-PEG-Mal polymer located on the particle surface. Conjugation with NHS-PEG-Mal to the surface of chitosan-coated particles contributed to steric stabilization, improved hemocompatibility, and introduction of functional groups for subsequent conjugation with iRGD peptide. iRGD-PEG-NPs were characterized by penetration into cells by receptor-mediated endocytosis. In both in vitro and in vivo experiments, iRGD-PEG-NPs induced a higher degree of colon tumor cell apoptosis than non-modified PEG-NPs. The in vivo experiment results showed that iRGD-PEG-NPs inhibit tumor growth and have good biocompatibility with animal organisms [93].

Another intriguing approach is the use of erythrocytes to coat polymer nano-scaled carriers. Erythrocytes, the most common blood cells with a long lifespan, are used to reduce the immunogenicity of the resulting system, avoid its capture by macrophages, and increase preparation circulation time in the organism [148]. Thus, mPEG5K-PCL20K-based nanoparticles loaded with icariin and coated with iRGD-modified erythrocyte membrane (iRINP) showed improved biocompatibility and stability and helped to avoid phagocytic uptake by macrophages. iRGD induced apoptosis and inhibited lung cancer cell migration, proliferation, and invasion in vitro. With good biocompatibility, iRINP reduced tumor size and inhibited tumor growth without side effects in vivo [149].

It should also be mentioned that polymer nanoparticles can deliver biologically active molecules bound onto their surface. Thus, plasmid DNA LHPP (phospholysine phosphohistidine inorganic pyrophosphate phosphatase), which is a tumor suppressor, was electrostatically bound to the surface of a nanoparticle obtained by self-assembly of C18-PEG-iRGD, monomethoxy poly(ethlene glycol)-poly(D, L-lactide) (mPEG-PLA), and N-[1-(2,3-dioleoyloxy) propyl]-N, N, N-trimethylammonium chloride (DOTAP). Despite the location of the plasmid DNA on the surface, the nanoparticle protects the plasmid from enzymatic cleavage. In vitro experiments showed that the iDPP/LHPP nanocomplex arrests the melanoma cell cycle in the G0/G1 phase and induces apoptosis through caspase-3 activation. Indeed, it was recently established that such nanoparticles successfully deliver LHPP to melanoma cells, inhibiting tumor growth in vivo without causing significant side effects [150].

However, although polymer nanocarriers are one of the most promising systems for delivering drugs into the body, the list of biocompatible polymers that lack immunogenicity is relatively limited. Other disadvantages to the introduction of some types of polymer nanoparticles worth mentioning could be side toxicity, non-optimal biodistribution in the body, insufficient efficiency, difficulty of production, or high cost.

### 4.4. iRGD Toxicity and Immunogenicity

As discussed above, CPPs are widely used as delivery tools to facilitate the intracellular transport of various cargoes, such as drugs, nucleic acids, and imaging agents. However, their clinical application is influenced by potential toxicity and immunogenicity concerns. The toxicity of CPPs is correlated with their structural properties and the mechanisms by which they interact with cell membranes. Indeed, cell–CPP interactions may disrupt cellular integrity, cause membrane destabilization, or trigger off-target effects. The degree of toxicity often varies based on CPP concentration, sequence composition, and the nature of the cargo.

The immunogenicity of CPPs is another critical point to consider, as the immune system may recognize certain CPPs as foreign. The recognition can result in adverse immune reactions, reducing CPP efficacy and potentially resulting in hypersensitivity or inflammatory responses. Modifications in CPP sequence, such as altering amino acid composition or using non-natural amino acids, have been explored to minimize immunogenicity and toxicity while preserving delivery efficacy [151,152].

iRGD, a tumor-penetrating peptide often used to enhance drug delivery specifically to cancer cells, typically exhibits lower systemic toxicity compared to many traditional CPPs. Unlike CPPs that are more indiscriminately internalized by cells, iRGD has a tumor-homing property due to its affinity for αvβ3 and αvβ5 integrins, which are overexpressed in the tumor microenvironment. This selectivity allows iRGD to concentrate in cancerous tissue, reducing off-target effects and thereby minimizing toxicity to healthy cells [153].

Indeed, in various studies, the iRGD peptide has been shown to increase the accumulation and penetration of anticancer drugs into tumors but not into normal tissues [1,11,136]. To date, iRGD-based drugs are already undergoing preclinical studies. In the first phase of an open-label, multicenter clinical trial by Lisata Therapeutics Inc., an iRGD-based drug called CEND-1 (also known as LSTA1) was tested in patients with metastatic pancreatic ductal adenocarcinoma. The study did not reveal dose-limiting toxicities for CEND-1. The most common adverse events were neutropenia, anemia, leukopenia, and pulmonary embolism. Patients experienced serious adverse events, which were mostly related to disease progression [76].

Additionally, the Phase 1b/2a CENDIFOX trial in the U.S. is evaluating the addition of LSTA1 to neoadjuvant FOLFIRINOX (eucovorin, 5-fluorouracil, and irinotecan) with or without panitumumab (Vectibix) in patients with pancreatic, colorectal, and appendiceal cancer [154]. The FDA also granted Orphan Drug Designation to the drug candidate LSTA1 (CEND-1) as a potential therapeutic option in patients with malignant glioma [https://tinyurl.com/2pvvuaja (accessed on 8 October 2024)].

In contrast, many CPPs, such as TAT (trans-activator of transcription)- or polyarginine-rich peptides, can cause higher cellular toxicity due to their strong, non-specific interactions with cell membranes. These interactions may destabilize the membrane or lead to excessive uptake by non-target cells, increasing the risk of cytotoxicity at therapeutic doses [151]. Thus, the iRGD’s targeted mechanism of action is related to a safer toxicity profile compared to other, more universally internalized CPPs, especially for applications requiring specific delivery to tumor cells [153].

## 5. Implementation of Artificial Intelligence

### 5.1. Implementation of Artificial Intelligence (AI) in Developing iRGD-Conjugated Therapeutics

The advancement of targeted cancer therapies has been significantly facilitated by introducing peptides like iRGD, designed to target tumor tissues selectively. iRGD-conjugated therapeutics have shown great potential in improving drug delivery to tumors, thereby enhancing efficacy and reducing systemic toxicity. However, optimizing these therapeutics requires addressing complicated challenges, including peptide design, drug conjugation, and predicting pharmacokinetic and pharmacodynamic properties. Integrating artificial intelligence (AI) in new therapeutics development is key to enhancing therapeutic effectiveness and uncovering new insights into the biological mechanisms at play, furthering the potential of iRGD-conjugated therapeutics.

#### 5.1.1. AI in Peptide Design and Optimization

The design and optimization of the iRGD peptide itself is one of the fundamental steps in developing iRGD-conjugated therapeutics. With its specific sequence of amino acids (CRGDKGPDC), this peptide binds to αβ3/β5 integrins on the surface of tumor cells, facilitating the penetration of drugs into the tumor microenvironment [11]. Furthermore, the development of cell adhesion peptides (CAPs), which are improved but still similar to RGD peptides, is another important research direction. However, discovering new CAPs for various applications is often complicated and time-consuming. In order to overcome this challenge, Wu et al. introduced an efficient computational pipeline that integrates sequence embeddings, binding predictors, and molecular dynamics simulations to streamline the discovery process. A Pro2vec model, trained on extensive CAP datasets, was used to identify tripeptide candidates similar to RGD. The binding affinity of identified candidate peptides with integrin receptors was, in continuation, assessed using the Mutabind2 ML model. Molecular dynamics simulations (MDSs) were also conducted to model receptor–peptide interactions and calculate binding free energies, providing a quantitative assessment for further screening. The selected peptide demonstrated similar performance to RGD in endothelial cell adhesion and spreading assays, confirming the effectiveness of this computational approach [155]. Indeed, computational tools have been utilized to develop other integrin-binding peptides [156].

A novel method for segmenting protein sequences into commonly occurring variable-length sub-sequences uses peptide-pair encoding (PPE). This approach was inspired by the byte-pair encoding (BPE) algorithm used in text compression. PPE is particularly useful in protein bioinformatics, offering a flexible representation for various ML tasks. The method was applied to integrins and integrin-binding proteins, demonstrating its effectiveness in protein motif discovery through the DiMotif tool. DiMotif reliably identified integrin-related motifs with high recall and F1 scores, making it valuable for detecting integrins, integrin-binding proteins, and biofilm-related proteins [157]. Moreover, deep learning (DL) methods have so far been used to facilitate the development of oligopeptide drugs, mainly focusing on integrin interactions. Researchers used an unsupervised DL model to identify patterns in intrinsically disordered regions of approximately 171 osteogenic proteins, leading to the generation of oligopeptides via Monte Carlo simulation. This line of research identified the AIB5P oligopeptide, which was found to significantly promote bone formation in various mouse models, including osteoporosis and fracture repair. Mechanistically, AIB5P enhances osteogenesis by binding to the integrin α5 subunit, thereby activating FAK signaling. Notably, this study established a DL-based strategy for oligopeptide discovery, showcasing its efficacy from early screening through in vivo validation.

The inclusion of additional binding domains is another research issue. Indeed, recently, glycoengineering techniques were recently utilized to synthesize a programmed cell death protein-1 (PD-1) antibody–iRGD cyclic peptide conjugate (αPD-1-(iRGD)2). This conjugate not only targets the RGD sequence on integrins but is specific for targets on immune cells, thus improving tissue penetration and engaging both tumor cells and PD-1+ T cells through dual targeting. As a result, it facilitates tumor-specific T-cell activation and proliferation while exerting minimal effects on non-specific cells [158].

#### 5.1.2. AI in Drug Conjugation and Formulation

AI has been instrumental in optimizing the conjugation chemistry. Computational models can predict the stability and release profile of an iRGD–drug conjugate by simulating various conjugation strategies. Factors such as the tumor’s chemical environment, the linker’s nature, and the interaction between the peptide and the drug are considered and assessed by AI-generated models. Furthermore, these models can identify potential issues, such as premature drug release or degradation, allowing for the design of more robust conjugates before in vivo application. The next challenge is to conjugate the iRGD peptide with the therapeutic cargo to preserve both the peptide’s targeting ability and the drug’s activity. Therefore, a suitable linker must be identified to ensure the drug remains stable in circulation and achieve simultaneously controlled release at the tumor site [159].

In addition to simulation, AI is also used in high-throughput screening of iRGD–drug conjugates. Machine learning algorithms can analyze data from large-scale experiments to identify patterns and correlations that might not be evident through manual analysis. The specific approach enables the rapid identification of conjugates with the most promising therapeutic profiles, minimizing the time and expense associated with experimental trial and error.

#### 5.1.3. AI in Predicting Pharmacokinetics and Pharmacodynamics

The pharmacokinetic (PK) and pharmacodynamic (PD) properties of iRGD-conjugated therapeutics are critical determinants of their success in clinical applications. PK refers to the drug’s absorption, distribution, metabolism, and excretion, while PD concerns the drug’s biological effects on the body. Traditional methods of predicting PK/PD properties often involve extensive in vivo studies, which are time-consuming and resource-intensive.

AI can transform this aspect of drug development by providing accurate predictions based on preclinical data. Machine learning models, including support vector machines and random forests, can be trained on datasets comprising the drug’s physicochemical properties, in vitro data, and previous in vivo results. These models can then predict how a new iRGD-conjugated therapeutic will behave in the body, including its distribution to the tumor, clearance rate, and potential off-target effects.

As a result, targeting strategies to enhance tumor and endothelial cells’ selective uptake of nanoparticles (NPs) have been devised. Notably, recent experiments validate the effectiveness of these nanoparticles. Thus, in a recent study, advanced molecular dynamics (MD) simulations and ML techniques were used to explore these interactions, focusing on a cyclic-RGD-conjugated PEGylated TiO_2_ NP and its binding with the extracellular segment of integrin αvβ3. Notably, the cyclic-RGD ligand was shown to maintain stable binding to the integrin even when attached to the NP. This approach, however, demonstrated chemical differences in binding the integrin when the cyclic RGD was attached to NP. Additionally, unbound cyclic RGDs on the NP contribute significantly to its interaction with the integrin. The binding affinity of the NPs is directly proportional to the density of cyclic RGDs on the NP, offering a venue for targeting selectivity and cellular uptake, which could lead to better clinical outcomes [160].

Moreover, AI can integrate various data types—such as genomic, proteomic, and metabolomic data—to provide a more comprehensive understanding of the drug’s PD effects. For instance, AI can help identify biomarkers that predict a patient’s response to the iRGD-conjugated therapeutic, which benefits personalized treatment approaches. This is particularly important in cancer therapy, where the tumor’s genetic makeup can significantly influence treatment outcomes [161].

AI is also being used to optimize dosing regimens for iRGD-conjugated therapeutics. By modeling the relationship between dose, exposure, and response, AI can recommend dosing strategies that maximize therapeutic efficacy while minimizing toxicity. These models can be continuously updated with new clinical data, ensuring that the dosing regimen remains optimal throughout the course of treatment [162,163].

#### 5.1.4. AI in Understanding Tumor Microenvironment and Mechanisms of Action

As discussed above, the TME is remodeled, with mechanical properties different from those of healthy tissues. The interaction of any therapeutic with the TME affects its penetrability and efficacy. AI can be used to model the interaction between the iRGD-conjugated therapeutic and the TME. Precisely, AI can model how the drug penetrates the tumor, how it is taken up by endothelial and cancer cells, and how the TME influences drug efficacy. The detailed understanding of these processes obtained from AI can guide the development of strategies to overcome barriers to drug delivery, such as dense ECMs or hypoxic regions within tumors [164,165].

#### 5.1.5. AI in Clinical Development and Personalized Medicine

Moreover, AI-driven approaches are paving the way for the utilization of iRGD-conjugated therapeutics in personalized medicine. Thus, AI can identify which patients are most likely to benefit from a particular iRGD-conjugated therapy by analyzing patient-specific data, such as genetic mutations, tumor characteristics, and immune profiles. This enables the personalization of treatment plans, improving outcomes and reducing unnecessary exposure to ineffective therapies.

**Figure 7:**
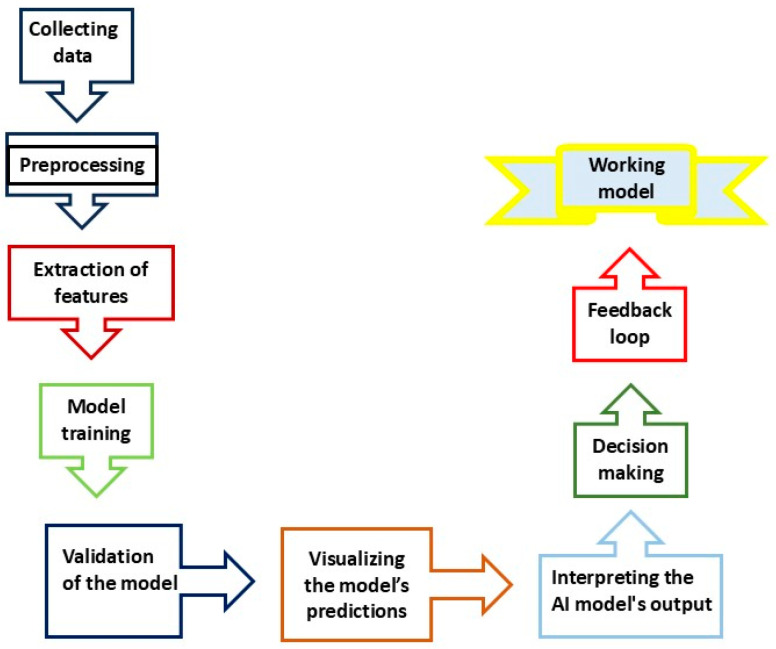
An outline of a workflow of AI utilization in peptide discovery and improvement for tumor targeting. The process involves gathering data on existing peptides, including their sequences, structures, interactions with tumor cells, and experimental results. The data are then cleaned, standardized, and preprocessed. Features such as amino acid composition and physicochemical properties are extracted. AI models are trained to predict effective peptide sequences and validated using known peptides. Visualization of predictions helps compare peptide efficacy, and interpretation guides decisions on which peptides to synthesize and test. The feedback loop integrates experimental results to refine the model, resulting in an AI tool capable of generating or improving tumor-targeting peptides ready for research or clinical use.

The iterative nature of this process is critical to achieving accurate predictions and effective peptide development. The application of AI facilitates resolving various challenges in the development of these therapies. As the utilization of AI technology continues to evolve, its role in optimizing and personalizing iRGD-conjugated therapeutics will undoubtedly expand, bringing us closer to more effective and safer cancer treatments. Figure 7 depicts the flowchart presenting the steps in obtaining AI-based working models for generating or improving CAPs.

## 6. Future Perspectives

As discussed above, iRGD has been attributed with a significant potential for use in the treatment of malignant disease. The advantages of iRGD are its simplicity, low cost of synthesis, and ease of nanoparticle modification, proteins, etc. However, the existing commonly used modification methods, such as NHS/EDC coupling and Michael reaction, have been poorly studied in relation to the iRGD peptide and can change the activity of the peptide, leading to its oligomerization and complete loss of activity. Although combined with iRGD, drugs, nanoparticles, and proteins can be effectively delivered to the tumor site, which reduces side effects, modification methods, and their effect on the activity of the peptide should also be studied. Currently, the iRGD peptide is widely studied for use in diagnosing and treating solid tumors, including in preclinical studies. Importantly, further safety profile studies should be conducted.

## 7. Conclusions—Summary

Chemotherapy, even though central to cancer treatment, often presents limited effectiveness due to inadequate drug penetration into solid tumors, immediately correlated to further tumor growth and dissemination. CPPs offer a solution by efficiently crossing cell membranes and delivering therapeutic agents with minimal toxicity. The iRGD peptide, in particular, is distinguished by its ability to target tumor-specific integrins (ανβ3/ανβ5) and neuropilin receptors, enabling precise, deeper penetration into tumor tissues. This dual-receptor targeting improves blood vessel permeability and enhances the delivery of co-administered drugs or nanoparticles. Taking into account the iRGD mechanism of action, application to different drug delivery systems, and future perspectives on iRGD-conjugated therapies, it can be concluded that it presents a promising application for improving treatment efficacy and reducing side effects.

## Figures and Tables

**Table 1:** Types of nanoparticles conjugated to iRGD.

Carrier	Type of Peptide Immobilization	Cancer Type	Loaded Drug	Refs.
Polymeric nanoparticles				
Polylactic acid	Michael reaction	Hepatocellular carcinoma	Vandetanib	[94]
PLGA	Michael reaction	Colon cancer	Camptothecin	[93]
Chitosan surface-modified poly (lactide-co-glycolides) nanoparticles	Michael reaction	Glioma	Carmustine and O^6^-benzylguanine	[107]
Caprolactone-12-hydroxystearic acid copolymer	NHS/EDC coupling	HepG2 line	Sorafenib and cisplatin	[96]
PLGA	NHS/EDC coupling	Rat colorectal carcinoma	Garcinol	[56]
PLGA	NHS/EDC coupling	Choroidal Neovascularization	microRNA-539-5p	[108]
Polysialic acid	NHS/EDC coupling	Colorectal cancer cells	Doxorubicin	[109]
Dendrimers				
PEGylated polyamidoamine	Acid-sensitive cis-aconite linkage	Brain tumor	Doxorubicin	[100]
Poly(amidoamine) dendrimer	N/A	Glioblastoma	Arsenic trioxide	[101]
Nanocage				
Gold nanocages	NHS/EDC coupling	Breast cancer	Doxorubicin	[103]
Protein nanocage	iRGD domain is attached to the C-terminal region of the HSP cage utilizing flexible linker moieties (30 amino acid linkers, (GGS)10)	Pancreatic cancer	OSU03012 (celecoxib derivative)	[102]
Inorganic nanoparticles				
Iron oxide nano-worms	Michael reaction	Breast cancer; brain metastasis	N/A	[104]
Gold nanoparticles	Michael reaction	Lung adenocarcinoma	siCDK	[110]
MoS2/Fe nanoparticles	NHS/EDC coupling	Triple-negative breast cancer	CPT-11	[111]
Cell-based nanocarriers				
Red blood cell	Michael reaction	Gastric cancer	N/A	[112]
Cytokine-induced memory-like natural killer	Michael reaction	Hepatocellular carcinoma	N/A	[113]
iRGD-antiCD3-modified T cells	Mixing	Gastric cancer	N/A	[114]
Polymeric micelles				
DSPE–PEG/LA–PLGA–TPGS mixed micelles	Michael reaction	Human cervical cancer line	Docetaxel	[115]
Polyethylene glycol-poly(2-(diisopropylamine)ethyl methacrylate-based polymeric micelles	NHS/EDC coupling	Glioma	Temozolomide and oleic acid-modified manganese oxide	[116]

## Data Availability

No new data were created.
